# Involvement of Nail Apparatus in Pemphigus Vulgaris in Ethnic Poles Is Infrequent

**DOI:** 10.3389/fmed.2018.00227

**Published:** 2018-08-14

**Authors:** Pawel Pietkiewicz, Monika Bowszyc-Dmochowska, Justyna Gornowicz-Porowska, Marian Dmochowski

**Affiliations:** ^1^Department of Dermatology, Poznan University of Medical Sciences, Poznan, Poland; ^2^Surgical Oncology and General Surgery Clinic I, Greater Poland Cancer Center, Poznan, Poland

**Keywords:** pemphigus vulgaris, nails, desmoglein 3, desmoglein 1, immunofluorescence, paronychia, onychomycosis, orifices

## Abstract

Pemphigus vulgaris lesions have a tendency to localize around natural body orifices. The aim here was to analyze the involvement of nail apparatus in pemphigus vulgaris. Sixty seven ethnic Poles suffering from pemphigus vulgaris on photographic files archiving initial presentation were retrospectively evaluated. Pemphigus vulgaris was diagnosed using combination of clinical data, H+E histology, direct immunofluorescence of plucked scalp hair and/or perilesional tissue also for IgG1 and IgG4 deposits evaluation, indirect immunofluorescence on mosaic substrate and/or monkey esophagus, mono-analyte ELISA with desmoglein 1/3 or multi-analyte ELISA. The nail apparatus involvement was found in 9 of 67 patients (13.4%; 3 females and 6 males). Periungual fingernail lesions were found in 6 patients (2 females, 4 males), whereas periungual toenail lesions in just 3 patients (1 female, 2 males). Our patients nail apparatus changes included, by order of frequency, paronychia, nail discoloration, onychorrhexis, Beau lines, periungual hemorrhages, onychomadesis, cross-ridging, onycholysis, and trachyonychia. The average time between the onset, as recalled by patients, and the diagnosis of pemphigus vulgaris with direct immunofluorescence was not statistically different in PV patients with and without nail apparatus lesions. In this article the molecular and immunological rationale for of periungual involvement is discussed. Our single-center study suggests that nail apparatus involvement is infrequent in pemphigus vulgaris in ethnic Poles. Due to the fact that nail apparatus lesions in pemphigus vulgaris may clinically resemble onychomycosis, giving the proper diagnosis can be difficult particularly when other lesions are overlooked or misinterpreted.

## Introduction

Pemphigus vulgaris (PV) is the most common constitute of pemphigus group of autoimmune blistering dermatoses (ABD). Although relatively rare, it is a severe, potentially life-threatening condition of a 3-fold increased risk of death than normal population (1). Scant literature data indicate yearly PV incidence as ranging between 0.076 and 1.6 per 100.000 persons (2, 3), yet it seems to be varied geographically, ethnically, and sex-dependent (with woman predominance noted in some studies) (3–7). While considered a disease characteristic for quintagenarians, it may occur at any age including childhood (7, 8).

There are two main forms of clinical manifestation of PV. In mucocutaneous PV (mcPV), pathological autoimmunity targets desmoglein 3 (DSG3; abundantly expressed in basal and parabasal layers of the epidermis and the mucosa) and desmoglein 1 (DSG1; abundantly expressed in upper layers of the epidermis but scantly expressed in the mucosa), whereas in mucosal dominant PV (mdPV)—DSG3 is classically targeted (9). Painful flaccid intraepidermal/suprabasal blisters in PV, lead to oozing, crusted, usually superinfected erosions. Nevertheless, in some patients no link between autoantibody profile and non-stereotypical clinical manifestation was noted, that led to distinction of, the so called, atypical variant of PV (e.g., cutaneous PV[cPV] with anti-DSG3 or anti-DSG1/3 IgG autoantibodies) (10, 11).

Nowadays, immunopathological studies (direct and indirect immunofluorescence; DIF, IIF) and enzyme-linked immunosorbent assay (ELISA) are regarded indispensable diagnostic tools in ABD diagnostics (12, 13). There are commercially available serological assays designed for precise target antigen identification, including novel biochip mosaic-based IIF and bioplex-based techniques (14–17). Moreover, some kits can be modified for specific IgG4 autoantibody detection and the identification of an active, Th-2 mediated stage of the disease (18, 19). IIF study on monkey esophagus in PV reveals circulating IgG/IgG4 class of pemphigus-type autoantibodies against desmosomal proteins of keratinocytes. Classic DIF of perilesional skin/mucosa in PV shows “fishing-net”/“honeycomb”/“basket weave”/“chicken wire” pattern (intercellular IgG+/-C3 deposits) (20) and “dew drops on spider web” appearance (punctate/granular intercellular IgG4 deposits) (21). For low-invasive diagnosis—perilesional skin can be substituted with plucked hair (21).

Although oral mucosa seems to be primarily affected in 50–70% of cases (20) due to increased density of DSG3 containing desmosomes, PV mucosal blisters and erosions also typically have a tendency to involve certain areas characterized by transitive epithelia—e.g. nasopharynx, external ear canal, conjunctivae, tear canals, lids, vermilion, armpits, groins, areolae, esophagus, scalp (hair follicles as a natural body orifices), navel, nails, anus, genitourinary mucosa of vagina/labia, urethra and penis/preputium. Transitional epithelium may be a focus of autoimmune/autoinflammatory process regardless of whether the orifices are natural or not (scars, fistulas) (22–24). Thus, if these lesions are isolated, they may mimic many diseases (mostly infectious or neoplastic) and pose a significant diagnostic challenge for a non-dermatologist (25). Although PV patients occasionally present nail apparatus involvement, there are scant studies thoroughly investigating this topic and even fewer utilizing statistical methods. The aim of this single-center, retrospective, observational study was to analyze the PV nail apparatus involvement in ethnic Poles.

## Materials and methods

This work was a part of studies approved by the local Ethical Committee of the Poznan University of Medical Sciences in Poland and informed written consent was obtained from each individual.

We analyzed retrospectively photographic archives (showing initial patients' fingernail/toenail involvement before the treatment) and medical charts of 67 ethnic Slavs, i.e., Poles (29 males, 38 females) suffering from PV recorded at the Department of Dermatology, Poznan University of Medical Sciences (Poznan, Poland) in the years 2002-2017. The diagnoses were based on the combination of clinical data, H+E histology, and DIF of plucked scalp hair and/or perilesional tissue also for IgG1 and IgG4 deposits evaluation, gradually introduced/changing diagnostic tools within assessed period: IIF on mosaic substrate and/or monkey esophagus (Euroimmun, Germany or MBL, Japan), mono-analyte ELISA with DSG1/3 (Euroimmun, Germany or MBL, Japan) or multi-analyte ELISA with envoplakin, type VII collagen, DSG1, DSG3, BP180, BP230 (Euroimmun, Germany). Paraneoplastic pemphigus patients were excluded from the study based on the laboratory/clinical/imaging findings and immunofluorescence studies. Bacteriological and fungal cultures as well as direct mycological KOH tests were performed to exclude infections in every case of nail involvement. Due to cost-effectiveness no nail clipping or PAS staining was performed. Nail apparatus involvement was statistically assessed concerning sex (n = 67), sites (n = 9)(fingernails/toenails) differences and PV subtype (n = 44) with Fisher exact test (CI 0.95). In 51 cases of PV (9 with nail apparatus involvement, 42 without nail involvement) we compared the time between the onset of the disease and the diagnosis made with DIF (weeks till diagnosis, wtD) with Mann-Whitney U test with correction for continuity (CI 0.95). The comparison of the wtD in mcPV and mdPV subtypes was assessed with Mann-Whitney U test with correction for continuity (CI 0.95), whereas the comparison of wtD in mcPV, mdPV, cPV subtypes was evaluated with Kruskal-Wallis test (CI 0.95). All statistical tests were performed using Statistica 12.0, (Tibco Software Inc., US).

## Results

**Figure 1:**
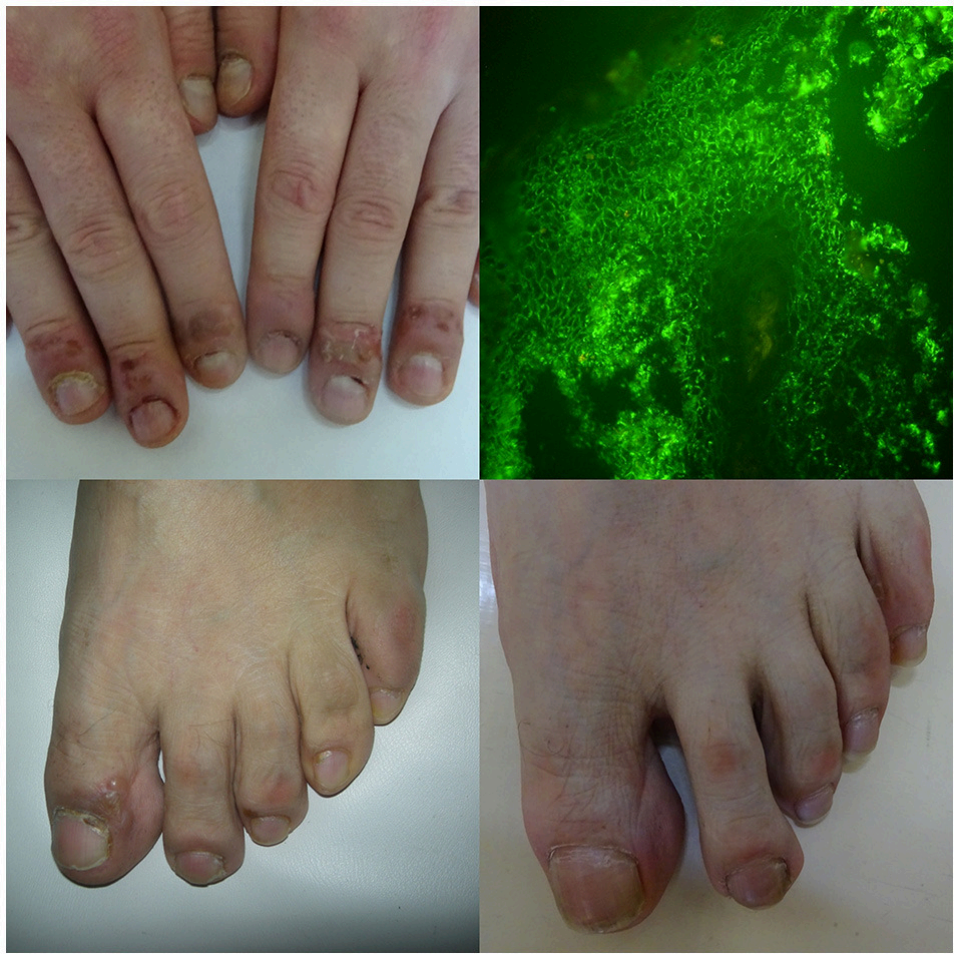
A young male, manual laborer, with mucocutaneous pemphigus vulgaris affecting also fingernails apparatus (featuring paronychia, onychomadesis, periungual hemorrhages, trachyonychia, Beau lines, and onychorrhexis), also suffering from onychotillomania and taking escitalopram for depression (**A**, upper panel left). Direct immunofluorescence of perilesional skin showed pemphigus intercellular IgG4 (++) deposits in both follicular and perifollicular epithelium having dew drops on spider web appearance (**B**, upper panel right) and multi-analyte ELISA revealed elevated levels of serum anti-DSG1 (value 1.8) and anti-DSG3 (value 4.4) IgG antibodies (cut-off values below 1). A middle-aged male with mucocutaneous pemphigus vulgaris having also toenails apparatus lesions (featuring paronychia, nail discoloration, onychorrhexis, and periungual hemorrhages)—before (**C**, lower panel left) and after immunosuppressive treatment (**D**, lower panel right). Direct immunofluorescence of perilesional skin showed pemphigus IgG1(+) and IgG4 (+ + +) intercellular deposits and mono-analyte ELISA revealed elevated levels of serum anti-DSG1 (>200 RU/ml and anti-DSG3 166.796 RU/ml) IgG antibodies (cut-off levels 20 RU/ml).

The nail apparatus involvement was found in 9 of 67 patients (13.4%; 3 females and 5 males with mcPV, 1 mdPV male) (Tables 1, 2 and Figure 1). Periungual fingernail lesions were found in 6 patients (2 females, 4 males), whereas periungual toenail lesions in just 3 patients (1 female, 2 males). None of the patients had concomitant fingernail and toenail lesions recorded in the archive. There were no significant differences in nail apparatus involvement neither between sexes (p=0.2460), sites (p=1.000) or PV subtypes (p=0.3891). There was no significant difference in wtD between PV patients with and without nail apparatus involvement (p=0.3126). No significant difference in wtD was observed either in mdPV and mcPV subtypes (p=0.3802) or between all the PV subtypes (p=0.3464).

**Table 1:** Nail apparatus involvement type and lesion location in ethnic Poles with pemphigus vulgaris (2002–2017).

**Nail apparatus involvement type**	**Number of patients with certain lesions (*n* = 9)**	**Fingernail involvement (males[m], females[f])**	**Toenail involvement (males[m], females[f])**	**Certain nail apparatus involvement differences regarding sites (fingernail vs. toenail; Fisher's exact test)**	**Certain nail apparatus involvement differences regarding sex (m vs. f; Fisher's exact test)**
Paronychia	9 (100%)	6 (66.67%) (5m, 2f)	3 (33.33%) (1m, 1f)	*p* = 1.000	*p* = 0.278
Nail discoloration	7 (77.78%)	4 (44.44%) (2m, 2f)	3 (33.33%) (2m, 1f)	*p* = 1.000	*p* = 0.456
Beau lines	5 (55.56%)	5 (55.56%) (3m, 2f)	0	*p* = 1.000	*p* = 0.645
Periungual hemorrhages	5 (55.56%)	3 (33.33%) (1m, 2f)	2 (22.22%) (2m)	*p* = 0.400	*p* = 1.00
Onychorrhexis	5 (55.56%)	2 (22.22%) (2m)	3(33.33%) (2m, 1f)	*p* = 0.400	*p* = 0.158
Onychomadesis	4 (44.44%)	4 (44.44%) (2m, 2f)	0	*p* = 1.000	*p* = 1.000
Cross-ridging	2 (22.22%)	2 (22.22%) (2m)	0	*p* = 1.000	*p* = 0.184
Onycholysis	1 (11.11%)	1 (11.11%) (1m)	0	*p* = 1.000	*p* = 0.433
Trachyonychia	1 (11.11%)	1 (11.11%) (1m)	0	*p* = 1.000	*p* = 0.433

**Table 2:** Average time between the PV onset and the diagnosis with DIF (wtD).

	**PV with nail apparatus involvement (*n* = 9)**	**PV without nail apparatus involvement (*n* = 52)**	**Mucocutaneous PV (*n* = 29)**	**Mucosal dominant PV (*n* = 13)**	**Cutaneous PV (*n* = 2)**
[-12pt] Average wtD (weeks)	27.78	23.60	22.86	23.85	56

## Discussion

The fingernails in PV are usually more affected than toenails (26, 27). Nail apparatus involvement may herald the recurrence and exacerbation of PV, while its intensity seems to be associated with the severity of the disease and area affected, with poor prognosis correlating with the presence of periungual/subungual/intraungual hemorrhages (26, 28–35). Periungual PV manifestations include paronychia, onychomadesis, onycholysis, Beau's lines, trachyonychia (rough nails), onychorrhexis (brittle nails), subungual hyperkeratosis, pterygium, nail dystrophy, nail discoloration, cross ridging, hemorrhagic nails, and periungual vegetating and verrucous lesions (31, 36–47). Our patients nail apparatus changes included, by order of frequency, paronychia, nail discoloration, onychorrhexis, Beau lines, periungual hemorrhages, onychomadesis, cross-ridging, onycholysis and trachyonychia. We found nail apparatus involvement in 13.4% of our PV patients. This is in contrast to the findings in previous studies from India (80%) (48), Iran (31.6%) (30), and USA (47%) (41) that showed higher prevalence. It is possible that ethnic and genetic differences between those populations and Polish one (e.g., haplogroups, HLA class II alleles) may be responsible for these odds. This hypothesis should be verified by further comparative studies in other European and Slavic populations, as this report seems to be the first in this ethnic group. Possibly, due to relatively low numbers of patients with PV nail apparatus involvement in our study, statistical analysis displayed no significant differences between sexes, sites and subtypes (Table 1). Although not statistically significant, in our study nail apparatus involvement was more frequent in males than in females in both sites (fingernails/toenails). Physical work pursued more likely by males due to sociocultural reasons may lead to higher tendency for traumatization, a known factor for developing PV lesions (42, 43). Unfortunately, having incomplete retrospective data about patients' occupation, we were not able to verify this presumption. The influence of male/female hormone balance modulating the inflammatory process at this specific site, may be another explanation of higher prevalence of PV nail involvement in man.

The most common symptoms of nail apparatus PV are paronychia (60%) and onychomadesis (30%) (31), what partially was also confirmed in our study (100, 44.44%, respectively). The most common causes of acute inflammation of the periungual folds include bacterial (*Streptococci, S. aureus*), viral (herpetic withlow) and fungal infections (*Fusarium, Candida, Neoscytalidum*), while drugs (including chemotherapy and targeted therapy) (49) and PV were reported to be the joint 4th most common cause (5% of all cases) (37, 50). Paronychia or paronychia mimics may also be triggered by several various pathologies: trauma (ingrown nail, nail biting, nail-sucking), parasitic infections, psoriasis (proximal nailfold psoriasis, acrodermatitis continua Hallopeau), neoplasms, and benign tumors. Onychomadesis is a state when the nail plate separates from the nail matrix, yet remains attached to the nail bed, that finally leads to nail plate shedding. Although the most common causes of onychomadesis are infections, severe illnesses, and drugs. The proper diagnosis may demand meticulous collection of medical history, examination and selection of accessory tests. Nail abnormalities in PV may not only succeed skin lesions and develop concomitantly with mucosal/skin lesions, but may also precede them (46, 51, 52). In such circumstances it may be a valuable hint easing the diagnostic process but also leading it astray and delaying the proper treatment. However, that process may be two-way. The diagnosis of PV should not discourage the physician from further investigation. Interestingly, 12% of already treated PV patients in Thai study, were found to have concomitant clinical onychomycosis (although only 5% were confirmed by mycological culture) (53). Similarly, an Egyptian study on PV female patients on immunosuppressive treatment also indicated 24% prevalence of onychomycosis, yet also 24% prevalence of bacterial periungual infection (54). What is worth noticing, fungal, and bacterial infection may mimic PV nail apparatus lesions and pose a threat to immunocompromised patients if left untreated, especially considering additional immunosuppressive treatment. Humidity of the periungual area (erosions, crusts, subungual hemorrhages) could be the predisposing factor for development of these infections in PV patients. Thus the hot climate of Egypt and Thailand may lead to relatively high incidence of infectious paronychia and onychomycosis, whereas the mild climate in Poland might be beneficial in that respect. Topical therapy was reported to be insufficient in nail manifestations of PV. Thus, systemic treatment is required to achieve nail recovery, usually with no subsequent nail deformation (31). All nail apparatus lesions in our PV patients responded to first-line immunosuppressive treatment schemes recommended for this disease with no subsequent nail involvement relapses, while oral lesions were commonly stubborn.

The precise mechanism leading to the initiation of pathological nail apparatus involvement in PV has not been elucidated. Hence, autoimmune process taking place in nail bed, matrix, and periungual nail fold could be explained by plausible complex associated with different expression of desmosomal cadherins in nail structures epithelia in comparison with skin and mucous membranes. Transitive stratified squamous epithelium could be regarded a target of immunization similarly as at other affected sites, what corresponds with acantholysis and PV patterns of IgG, IgG1, and IgG4 in DIF (21). Relative rarity of nail apparatus involvement is considered by some a consequence of partial sequestration of target antigens in proximal nail matrix (PNM) and reduced number of antigen presenting cells as well as depression of their functions in comparison to mucosal and skin immune system, but resembling the one of hair follicle (31, 55, 56). Moreover, apart from locally increasing levels of potent immunosuppressive cytokines, i.e., transforming growth factor-β1 (TGF-β1) and α-melanocyte stimulating hormone (α-MSH), PNM keratinocytes downregulate MHC class I, Langerhans cells downregulate MHC class II and CD209 expression, while numbers and functions of NK lymphocytes and mast cells in periungual area are also reduced giving the “immune privilege” conditions against autoimmunity (55, 56). Still, hairy skin seems to be preferentially affected by PV lesions compared to nail apparatus, which may suggest that the sheer surface size, and not “immune privilege,” is the critical factor determining appendageal sites of predilection. Nonetheless, compensation theory explains the involvement of these sites as areas supposedly more prone to anti-DSG3-directed autoimmunity and subsequent keratinocyte detachment without any relation to anatomical/structural morphology of the epithelium where low DSG1 expression is accompanied by compensatory higher expression of DSG3. Interestingly, this is not the case in nails, where both DSG1 and DSG3 are expressed at low level due to the absence of granular layer in nail matrix and thickness (2–3 cell layers) of nail bed epithelium (57). Thus, only high titers of anti-DSG1/anti-DSG3 or anti-DSG3 autoantibodies alone cause the collapsing of “immune privilege” and PV nail apparatus involvement (57, 58).

As nail apparatus involvement was not the sole presentation in any of our PV cases, we suspect that it is just a sign of a severe and protracting disease. We assumed that nail involvement in PV may encourage the patients to seek help earlier. Interestingly, wtD in patients with nail involvement was greater than in patients without nail involvement (Table 2), although the differences were not shown to be statistically significant. This ca. 1-month delay in periungual PV diagnosis may suggest that these symptoms might have been treated as a fungal/bacterial/viral infection by the physician or were regarded a minor/non-related issue either by the patient or a physician. On the other hand, it is possible that the periungual PV patients, driven by the embarrassment and lowered self-esteem were reluctant to seek professional help until full-blown PV developed. Further studies should be performed in this group to evaluate the seriousness of this burden concerning the social impact of PV. No subtype was diagnosed significantly faster than other (Table 2), however the assessment of vast range of symptoms and locations involved may be a limitation. Hence, this relation should also cover the aspect of severity of the disease subtype measured with uniform scoring system in the future studies. Relation between nail apparatus involvement in PV and IIF, ELISA, DIF was not assessed in this study due to incomplete database for all the patients, as in 2002–2017 diagnostic algorithms changed and new diagnostic tools were implemented (in our department multi-analyte ELISA superseded mono-analyte ones and we ceased using subjective imaging IIF), suppliers changed (different titration units) and some became redundant or were used occasionally as supplemental procedures. Further studies should provide more information on the association between nail involvement and severity of the disease.

It is concluded that nail apparatus involvement is infrequent in pemphigus vulgaris in ethnic Poles. Thus, nail apparatus lesions can be misleading when practicing dermatologists examine just periungual body areas that bother patients most, overlook, and/or misinterpret other lesions. Conversely, nail apparatus involvement, although embarrassing for the patient, can be an invaluable hint as to PV diagnosis when meticulously analyzed in the clinical context, such as symptoms evolution, medical histories including family history, concomitant malignancies (59), culprit medications (60), radiation (61), trauma (42) and airborne and topical chemical compounds exposure (61–63). Proper treatment, including cessation of triggering factors, can facilitate good control, and faster recovery, reduce the burden of aggressive treatment and prevent aggravation of PV, possibly the lethal disease.

## Author contributions

MD and PP contributed conception and design of the study. PP organized the database and performed the statistical analysis. PP and MD wrote the first draft of the manuscript. PP, MB-D, MD, and JG-P wrote sections of the manuscript. All authors contributed to manuscript revision, read and approved the submitted version.

### Conflict of interest statement

The authors declare that the research was conducted in the absence of any commercial or financial relationships that could be construed as a potential conflict of interest.

## Abbreviations

PV: pemphigus vulgaris
mcPV: mucocutaneous PV
mdPV: mucosal dominant PV
cPV: cutaneous PV
DIF: direct immunofluorescence study.
